# Inhibitors of ORAI1 Prevent Cytosolic Calcium-Associated Injury of Human Pancreatic Acinar Cells and Acute Pancreatitis in 3 Mouse Models

**DOI:** 10.1053/j.gastro.2015.04.015

**Published:** 2015-08

**Authors:** Li Wen, Svetlana Voronina, Muhammad A. Javed, Muhammad Awais, Peter Szatmary, Diane Latawiec, Michael Chvanov, David Collier, Wei Huang, John Barrett, Malcolm Begg, Ken Stauderman, Jack Roos, Sergey Grigoryev, Stephanie Ramos, Evan Rogers, Jeff Whitten, Gonul Velicelebi, Michael Dunn, Alexei V. Tepikin, David N. Criddle, Robert Sutton

**Affiliations:** 1 Pancreas Biomedical Research Unit, National Institute for Health Research Liverpool, Royal Liverpool University Hospital, Liverpool, United Kingdom; 2 Department of Molecular and Cellular Physiology, Institute of Translational Medicine, University of Liverpool, Liverpool, United Kingdom; 3 Department of Integrated Traditional and Western Medicine, Sichuan Provincial Pancreatitis Centre, West China Hospital, Sichuan University, Chengdu, People's Republic of China; 4 Respiratory Therapy Area Unit, Medicines Research Centre, GlaxoSmithKline, Stevenage, United Kingdom; 5 CalciMedica, La Jolla, California

**Keywords:** STIM1, SOCE, Calcium Entry Inhibition, Drug Development, Experimental Pancreatitis, AP, acute pancreatitis, [Ca^2+^]_C_, cytosolic calcium concentration, CCK, cholecystokinin, CER, cerulein, FAEE, fatty acid ethyl ester, IC_50_, half-maximal inhibitory concentration, I_CRAC_, Ca^2+^ release-activated Ca^2+^ currents, IL, interleukin, MPO, myeloperoxidase, PI, propidium iodide, SOCE, store-operated calcium entry, STIM, stromal interaction molecule, TLCS, taurolithocholate acid sulfate

## Abstract

**Background & Aims:**

Sustained activation of the cytosolic calcium concentration induces injury to pancreatic acinar cells and necrosis. The calcium release–activated calcium modulator ORAI1 is the most abundant Ca^2+^ entry channel in pancreatic acinar cells; it sustains calcium overload in mice exposed to toxins that induce pancreatitis. We investigated the roles of ORAI1 in pancreatic acinar cell injury and the development of acute pancreatitis in mice.

**Methods:**

Mouse and human acinar cells, as well as HEK 293 cells transfected to express human ORAI1 with human stromal interaction molecule 1, were hyperstimulated or incubated with human bile acid, thapsigargin, or cyclopiazonic acid to induce calcium entry. GSK-7975A or CM_128 were added to some cells, which were analyzed by confocal and video microscopy and patch clamp recordings. Acute pancreatitis was induced in C57BL/6J mice by ductal injection of taurolithocholic acid 3-sulfate or intravenous' administration of cerulein or ethanol and palmitoleic acid. Some mice then were given GSK-7975A or CM_128, which inhibit ORAI1, at different time points to assess local and systemic effects.

**Results:**

GSK-7975A and CM_128 each separately inhibited toxin-induced activation of ORAI1 and/or activation of Ca^2+^ currents after Ca^2+^ release, in a concentration-dependent manner, in mouse and human pancreatic acinar cells (inhibition >90% of the levels observed in control cells). The ORAI1 inhibitors also prevented activation of the necrotic cell death pathway in mouse and human pancreatic acinar cells. GSK-7975A and CM_128 each inhibited all local and systemic features of acute pancreatitis in all 3 models, in dose- and time-dependent manners. The agents were significantly more effective, in a range of parameters, when given at 1 vs 6 hours after induction of pancreatitis.

**Conclusions:**

Cytosolic calcium overload, mediated via ORAI1, contributes to the pathogenesis of acute pancreatitis. ORAI1 inhibitors might be developed for the treatment of patients with pancreatitis.

Sustained increase of the cytosolic calcium concentration ([Ca^2+^]_C_) is a critical trigger for pancreatic acinar cell injury and necrosis, which depends on store-operated calcium entry (SOCE).^1^, ^2^, ^3^, ^4^ ORAI1 is the principal SOCE channel in the pancreatic acinar cell,^5^ the opening of which is coordinated by stromal interaction molecule (STIM)1 and STIM2, after decreases in endoplasmic reticulum calcium store concentrations.^3^, ^5^, ^6^, ^7^ GSK-7975A and CM_128 were developed independently by GlaxoSmithKline (Stevenage, United Kingdom)^3^, ^7^, ^8^ and CalciMedica (La Jolla, CA), respectively, to block ORAI1 channels, although only CM_128 continues toward clinical development. GSK-7975A inhibits SOCE induced by thapsigargin in isolated murine pancreatic acinar cells over the range of 1–50 μmol/L (half-maximal inhibitory concentration [IC_50_], ∼3.4 μmol/L),^3^ inhibits endocytic vacuole formation^9^ and reduces necrosis induced by toxins that cause acute pancreatitis.^3^, ^9^ CM_128 is a new molecular entity. ORAI1 inhibition could inhibit SOCE and necrosis in human pancreatic acinar cells and ameliorate acute pancreatitis.

Genetic knockout of the transient receptor potential canonical 3 channel,^10^ a nonselective cation channel regulated in part by STIM1 via transient receptor potential canonical 1,^11^ resulted in an approximately 50% reduction of in vivo serum amylase increase and edema formation induced by 4 injections of cerulein.^10^ These experiments supported some role for SOCE in acute pancreatitis, but in a single mild model with few parameters of response.

Here, we defined the concentration-dependent inhibitory effects of GSK-7975A and CM_128 on SOCE and necrosis in murine and human pancreatic acinar cells induced by taurolithocholic acid 3-sulfate (TLCS)^2^, ^12^ or cholecystokinin (CCK) 8.^1^, ^10^ The effects of CM_128 on ORAI1 were substantiated by examination of its effect on Ca^2+^ release-activated Ca^2+^ currents (I_CRAC_)^3^, ^6^, ^7^ in ORAI1/STIM1-transfected HEK 293 cells.^7^ Our in vitro work informed in vivo pharmacokinetic analysis. GSK-7975A was given at selected doses after induction of acute pancreatitis (AP) with TLCS (TLCS-AP),^13^ 7 injections of cerulein (CER-AP)^14^ or ethanol and palmitoleic acid (FAEE-AP).^15^ Because GSK-7975A markedly reduced all parameters of pathobiologic response in a dose-dependent manner, a high dose of GSK-7975A and separately CM_128 was begun at 2 different time points after disease induction to determine the effect of early vs late drug administration. Drug administration that was begun 1 hour after disease induction was highly effective in reducing parameters of pathobiologic response, significantly more so than when begun 6 hours after disease induction, in all models. These data provide thorough preclinical validation for ORAI channel inhibition as a potential early treatment for acute pancreatitis.

## Materials and Methods

### Human Specimen Sampling

Human pancreas was sampled and cells were isolated as described.^16^ The time from sampling to the start of cell isolation was fewer than 10 minutes.

### Cell Culture and Transfection

HEK 293 cells were cultured and transfected as described.^7^ HEK 293 cells stably transfected with complementary DNAs encoding human ORAI1 and STIM1 were used in patch-clamp recording.

### Animals

CD-1 and C57BL/6J mice were from Charles River UK, Ltd (Margate, Kent, UK). Pancreatic acinar cells were isolated from CD-1 mice as described.^1^, ^3^, ^12^, ^15^ For in vivo experiments, 10-week-old male C57BL/6J mice (25 g) were used.

### Confocal Fluorescence Microscopy and Video Imaging

Isolated pancreatic acinar cells were imaged using a Till Photonics System (Munich, Germany) to assess [Ca^2+^]_C_ with Fura-2 (5 μmol/L; excitation, 340 and 380 nm; emission, >490 nm; ratio of fluorescence recorded from excitation, 340 and 380 nm) and using LSM710 systems (Carl Zeiss, Jena GmbH) to assess necrotic cell death pathway activation with propidium iodide (PI) (1 μmol/L; excitation, 488 nm; emission, 630–693 nm).

### Necrotic Cell Death Pathway Activation Measurement

Cells were treated with GSK-7975A or CM_128 together with TLCS (500 μmol/L) for 30 minutes, gently shaking at 1000 rpm at room temperature. After washing, cells were stained with PI and Hoechst 33342, distributed into 96-well glass bottom plates (150 μL/well), and imaged using LSM710 systems. Hoechst 33342 (50 μg/mL; excitation, 364 nm; emission, 405–450 nm) was used to stain nuclei and count the total number of cells. PI was used to assess plasma membrane rupture: the total number of cells showing PI uptake was counted in 3 or more wells and in 12 or more random fields of each differently treated group of each isolate to provide a percentage, averaged across fields, as the mean ± SEM field percentage PI uptake with 3 or more isolates per group, except where stated.

### Patch-Clamp Current Recording

The whole-cell configuration was used to record I_CRAC_ from hORAI1/hSTIM1 HEK 293 cells.^7^ Patch pipettes were pulled from borosilicate glass capillaries (Sutter Instruments) with a resistance of 2–5 MΩ when filled with an extracellular solution of 120 mmol/L NaCl; 10 mmol/L TEA-Cl; 10 mmol/L HEPES; 10 or 0 mmol/L CaCl_2_; 2 or 12 mmol/L MgCl_2_, and 10 mmol/L glucose, pH 7.2. I_CRAC_ was activated by passive depletion of intracellular Ca^2+^ stores using the intracellular solution of 105 mmol/L Cs-glutamate; 10 mmol/L HEPES; 20 mmol/L 1,2-bis(o-aminophenoxy)ethane-N,N,N',N'-tetraacetic acid; 8 mmol/L MgCl_2_, pH 7.2. Patched cells were exposed to Ca^2+^-free buffer to establish stable baseline (for 5 min), then 10 mmol/L CaCl_2_ to develop I_CRAC_ (for 10 min), and then CM_128 (0.001, 0.01, 0.1, and 1 μmol/L for 10 min). External recording saline with no Ca^2+^ then was perfused for 2 minutes to determine the background current in the absence of I_CRAC_. Whole-cell currents were sampled at 10 KHz and filtered at 2 KHz (Multiclamp 700B amplifier and PClamp software; Axon Instruments). The voltage clamp protocol included a cycle of steps to 0 mV (for 10 ms to evaluate zero current), then -100 mV (for 10 ms to measure I_CRAC_), and a ramp from -100 mV to +100 mV over 50 ms for I-V relationship followed by step to +50 mV (for 10 ms to estimate leak current). The voltage between sweeps was +30 mV (for 12 s). Whole-cell capacitive compensation was used. Data analysis was performed using Clampfit software. I_CRAC_ was measured at -100 mV and current was measured at approximately 6 minutes and was used as the baseline control. The current measured after a 10-minute application of test compound was normalized to the baseline current (expressed as the percentage of control). The current measured in zero Ca^2+^ buffer was used to subtract the background leak current. Data points were fitted by nonlinear regression analysis with variable slope (SigmaPlot software) to determine the IC_50_ and Hill slope. The IC_50_ was taken as the point on the nonlinear regression halfway between the extrapolated baseline (control) and maximum inhibition produced by the compound.

### Experimental Acute Pancreatitis

TLCS-AP was induced by retrograde pancreatic ductal injection with 3 mmol/L TLCS (5 μL/min over 10 minutes by infusion pump)^13^; humane killing was 6 or 24 hours later. CER-AP was induced by 7 hourly intraperitoneal cerulein injections (50 μg/kg)^14^; humane killing was 12 hours after the first. FAEE-AP was induced by 2 hourly intraperitoneal injections of 150 mg/kg palmitoleic acid and 1.35 g/kg ethanol^15^; humane killing was 6 or 24 hours later. GSK-6288B, the prodrug of GSK-7975A, was administered by minipump; CM_128 was administered by intraperitoneal injection (≥6 mice/group).

### Enzyme Activity and Interleukin 6 Measurement

Trypsin activity was measured as described^17^ in homogenized tissue (Boc-Gln-Ala-Arg-MCA substrate; excitation, 380 nm; emission, 440 nm). Responses without treatment were normalized to 100 with SEM to compare different doses of either drug at different time points across models. Serum amylase was determined by a Roche Analyzer (Roche); interleukin (IL)6 was determined by enzyme-linked immunosorbent assay (R&D Systems).

### Myeloperoxidase Activity

Myeloperoxidase (MPO) activity was determined as described.^18^ Pancreatic or lung tissue was homogenized, resuspended in 100 mmol/L phosphate buffer (pH 5.4) containing 0.5% hexadecyltrimethyl ammonium bromide, 10 mmol/L EDTA and protease inhibitors, freeze-thawed 3 times, sonicated for 30 seconds, and centrifuged for 15 minutes at 16,000 × g. MPO activity was measured in supernatants (3,3,5,5-tetramethylbenzidine substrate with 0.01% H_2_O_2_). Absorbance was measured at 655 nm and MPO was calculated as the difference between absorbance at 0 and 3 minutes.

### Histology

Pancreatic tissue was fixed in 10% formalin, embedded in paraffin, and stained (H&E). Evaluation was performed on 10 random fields (×200) by 2 blinded independent investigators grading (scale, 0–3) edema, inflammatory cell infiltration, and acinar necrosis, calculating the means ± SEM (≥6 mice/group).

### Chemicals, Reagents, and Minipumps

CCK-8 was from American Peptide; fluorescent dyes were from Molecular Probes; Boc-Gln-Ala-Arg-MCA was from the Peptide Institute (Osaka, Japan); protease inhibitors were from Roche GmbH (Mannheim, Germany); IL6 quantikine enzyme-linked immunosorbent assay kit was from R&D Systems; and other reagents were from Sigma (Dorset, United Kingdom). 2,6-difluoro-N-(1-(4-hydroxy-2-(trifluoromethyl)benzyl)-1H-pyrazol-3-yl)benzamide (GSK-7975A) and pro-drug GSK-6288B were a gift from GlaxoSmithKline. CM_128 was a gift from CalciMedica. ALZET osmotic mini-pumps (2001D) were from Charles River UK, Ltd.

### Statistical Analysis

Data are presented as means ± SEM. Comparisons were performed by the 2-tailed Student *t* test or chi-squared test, and *P* values less than .05 were considered significant.

### Study Approval

Human pancreatic samples were obtained with informed consent as approved by the Liverpool Adult Local Research Ethics Committee (ref: 03/12/242/A). All animal studies were ethically reviewed and conducted according to UK Animals (Scientific Procedures) Act of 1986, approved by the UK Home Office (PPL 40/3320, renewed as 70/8109).

## Results

### Effects of GSK-7975A and CM_128 on Human Pancreatic Acinar Cells

**Figure 1:**
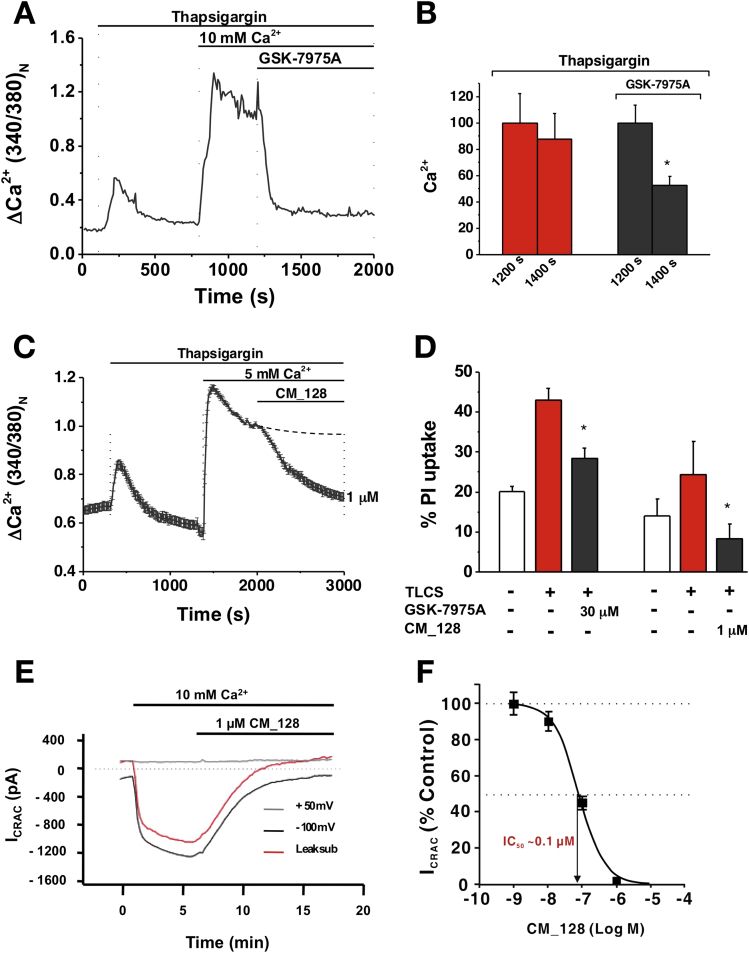
GSK-7975A and CM_128 inhibit CRAC entry (Fura-2 340:380 normalized at 1200 or 2000 s) and necrosis (PI uptake) in human pancreatic acinar cells and CM_128 concentration-dependently inhibits I_CRAC_ in hORAI1/hSTIM1 HEK 293 cells. (*A*) Typical trace showing the inhibitory effect of GSK-7975A (50 μmol/L) on thapsigargin-induced Ca^2+^ influx. (*B*) Mean (±SEM) [Ca^2+^]_C_ at 1200 and 1400 s from thapsigargin and thapsigargin plus GSK-7975A traces, showing a marked reduction with GSK-7975A (≥20 cells/group; *P < .001; thapsigargin vs thapsigargin plus GSK-7975A at 1400 s). (*C*) Changes in human pancreatic acinar [Ca^2+^]_C_ induced by thapsigargin (Fura-2 340:380 normalized at 2000 s), showing the inhibitory effect of 1 μmol/L CM_128. (*D*) GSK-7975A and CM_128 protected isolated human pancreatic acinar cells from necrotic cell death pathway activation induced by TLCS (500 μmol/L) (mean ± SEM; 3 experiments/group for GSK-7975A; *P < .05, TLCS vs TLCS plus GSK-7975A and 1 experiment/group [4 wells and 16 high-power fields each; total. 172 control cells, 97 TLCS, 110 TLCS, and CM_128] for CM_128; *P < .05, TLCS vs TLCS plus CM_128). (*E*) Typical trace showing I_CRAC_ current in response to Ca^2+^-depletion protocol with 1 μmol/L CM_128 in hORAI1/hSTIM1 HEK 293 cells. (*F*) Concentration-dependent inhibitory effects of CM_128 on I_CRAC_ current.

Potential translational applications of SOCE inhibition as a treatment for clinical acute pancreatitis were evaluated by examination of the effects of GSK-7975A or CM_128 on isolated human pancreatic acinar cells.^16^ Thapsigargin was used in zero external Ca^2+^ to empty Ca^2+^ stores, stimulate STIM-mediated Orai pore formation, and permit SOCE by the re-introduction of external Ca^2+^. GSK-7975A (10–50 μmol/L) inhibited SOCE in these cells (Figure 1*A* and *B*). CM_128 also was found to inhibit SOCE in human pancreatic acinar cells at lower concentrations (Figure 1*C*). Both GSK-7975A (30 μmol/L) and CM_128 (1 μmol/L) inhibited necrotic cell death pathway activation in these cells (Figure 1*D*). To verify that CM_128 inhibits human ORAI1, HEK 293 cells transfected with ORAI1/STIM1^7^ were patched in zero extracellular Ca^2+^ to measure I_CRAC_ in response to extracellular addition of 10 mmol/L Ca^2+^, and the effect of a range of concentrations of CM_128 tested. CM_128 was found to inhibit I_CRAC_ in a direct concentration-dependent manner, with IC_50_ at approximately 0.1 μmol/L and no loss of effect at high concentrations (10 μmol/L) (Figure 1*E* and *F*). These findings were confirmed over the same concentration range with FLIPR technology (data not shown).

### Effects of GSK-7975A on Murine Pancreatic Acinar Cells

**Figure 2:**
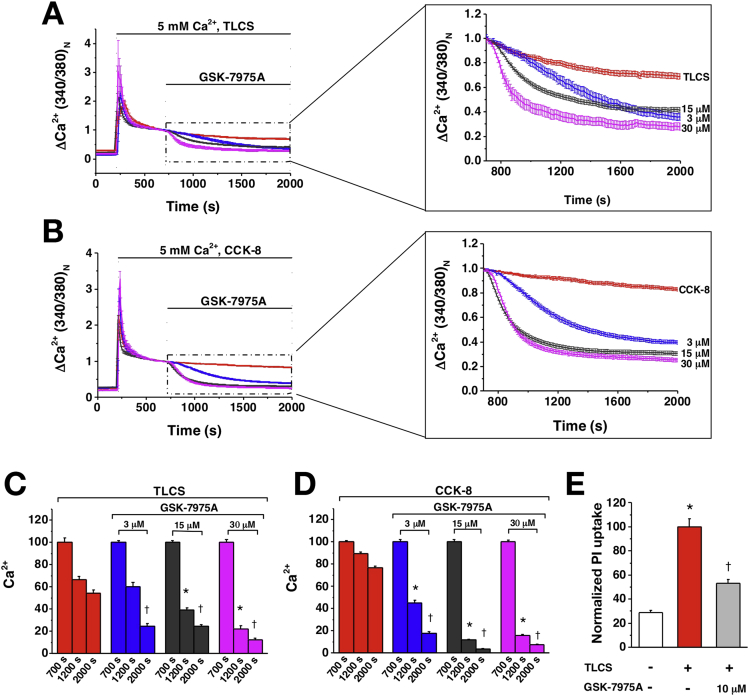
GSK-7975A concentration-dependently inhibits CRAC entry (Fura-2 340:380 normalized at 700 s) and necrosis (PI uptake). Changes in mouse pancreatic acinar [Ca^2+^]_C_ induced by (*A*) TLCS (500 μmol/L) and (*B*) CCK (1 nmol/L) showing effects of GSK-7975A from 700 s, expanded. (*C* and *D*) Mean (±SEM) [Ca^2+^]_C_ at 700, 1200, and 2000 s from panels *A* and *B*, showing progressive reduction with increasing GSK-7975A (≥19 cells/group; *P < .001, toxin vs toxin plus GSK-7975A at 1200 s; ^†^*P* < .001 at 2000 s). (*E*) GSK-7975A protected isolated murine pancreatic acinar cells from necrotic cell death pathway activation induced by TLCS (500 μmol/L) (mean ± SEM, normalized to TLCS at 100; ≥3 experiments/group; *P < .001, control vs TLCS; ^†^*P* < .001, TLCS vs TLCS plus GSK-7975A).

**Supplementary Figure 1:**
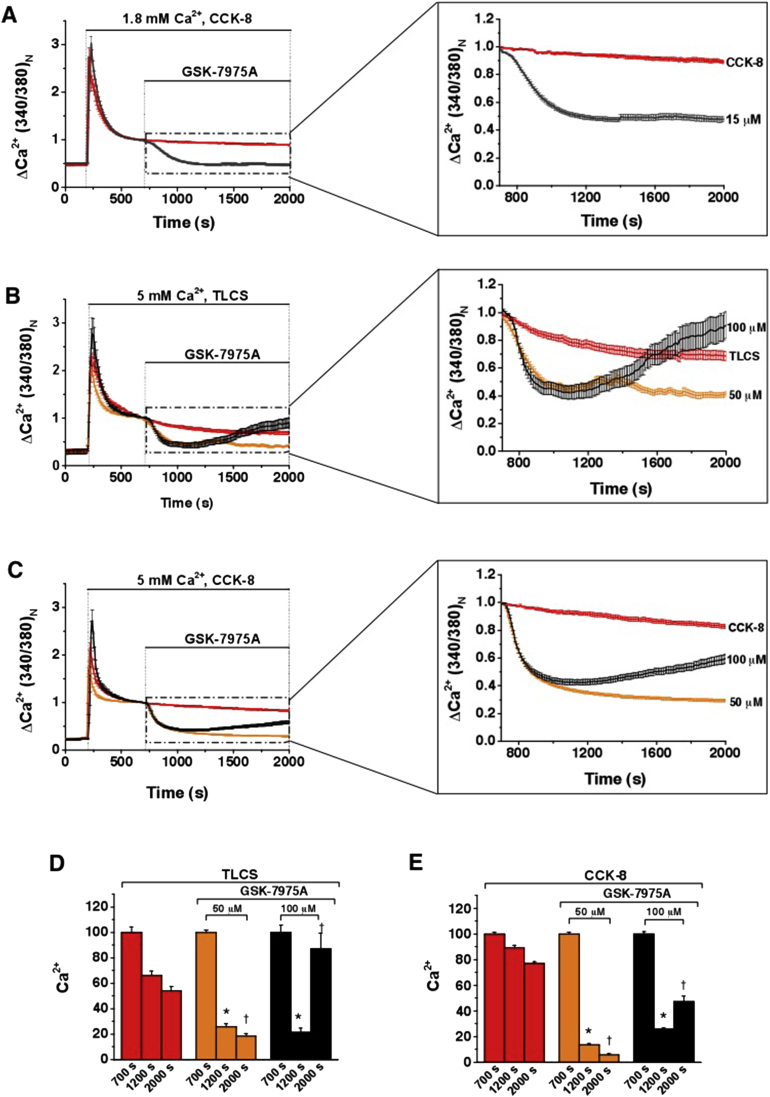
GSK-7975A inhibits CRAC entry (Fura-2 340:380 normalized at 700 s). (*A*) Changes in mouse pancreatic acinar [Ca^2+^]_C_ induced by CCK (1 nmol/L) with external physiological [Ca^2+^] (1.8 mmol/L) applied, showing the effect of 15 μmol/L GSK-7975A from 700 seconds, expanded (≥79 cells/group). Changes in mouse pancreatic acinar [Ca^2+^]_C_ induced by (*B*) TLCS (500 μmol/L) and (*C*) CCK (1 nmol/L), showing effects of 50 and 100 μmol/L GSK-7975A from 700 seconds, expanded. (*D* and *E*) Mean (±SEM) [Ca^2+^]_C_ at 700, 1200, and 2000 seconds from panels *B* and *C*, showing a marked reduction with 50 μmol/L GSK-7975A, but not 100 μmol/L GSK-7975A (≥27 cells/group; *P < .001, toxin vs toxin plus GSK-7975A at 1200 s; ^†^*P* < .001, at 2000 s).

Isolated murine pancreatic acinar cells maintained in 5 mmol/L external Ca^2+^ were perfused with TLCS (500 μmol/L) or supramaximal CCK (1 nmol/L) to induce sustained increases of [Ca^2+^]_C_ dependent on SOCE.^1^, ^3^, ^6^, ^10^ Once a stable plateau in [Ca^2+^]_C_ had formed, a range of fixed concentrations (0–100 μmol/L) of GSK-7975A were added. Increasing concentrations of GSK-7975A decreased the [Ca^2+^]_C_ plateau progressively and increasingly rapidly (Figure 2*A–D*). With TLCS, suppression of [Ca^2+^]_C_ toward the initial baseline approached 80% using 30 μmol/L GSK-7975A; with CCK, more than 95% using 15 μmol/L GSK-7975A, an effect also seen when cells were maintained in 1.8 mmol/L external Ca^2+^ (Supplementary Figure 1*A*). At 100 μmol/L GSK-7975A, but not at 50 μmol/L GSK-7975A, there was a loss of effect through an unknown mechanism (Supplementary Figure 1*B–E*). Necrotic cell death pathway activation was reduced markedly in murine pancreatic acinar cells by GSK-7975A (Figure 2*E*).

### Effects of CM_128 on Murine Pancreatic Acinar Cells

**Figure 3:**
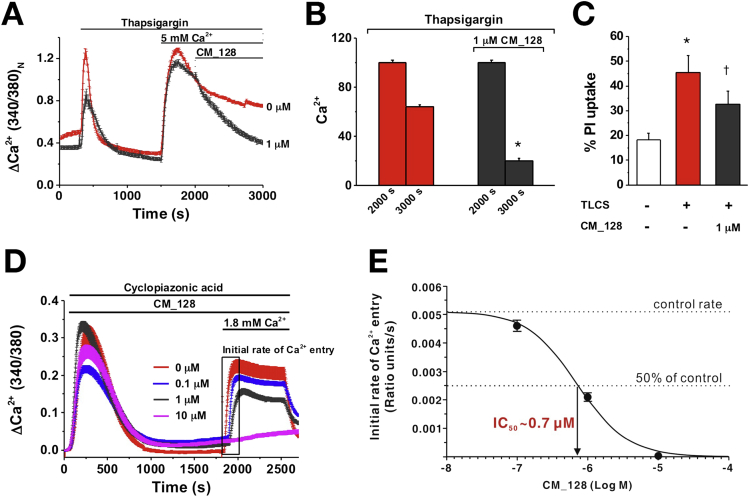
CM_128 concentration-dependently inhibits CRAC entry and necrosis (PI uptake). (*A*) Changes in mouse pancreatic acinar [Ca^2+^]_C_ induced by thapsigargin (Fura-2 340:380 normalized at 2000 s), showing effect of 1 μmol/L CM_128. (*B*) Mean (±SEM) [Ca^2+^]_C_ at 2000 and 3000 s from panel *A*, showing a marked reduction with 1 μmol/L CM_128 (≥62 cells/group; *P < .001, thapsigargin vs thapsigargin plus CM_128 at 3000 s). (*C*) CM_128 protected isolated murine pancreatic acinar cells from necrotic cell death pathway activation induced by TLCS (500 μmol/L) (mean ± SEM; ≥3 experiments/group; *P < .001, TLCS vs control; ^†^*P* < .05, TLCS vs TLCS plus CM_128). (*D*) Concentration-dependent inhibitory effects of CM_128 on cyclopiazonic acid–induced Ca^2+^ influx, showing a progressive reduction of the initial rate of Ca^2+^ entry and plateau with increasing CM_128, with complete inhibition of Ca^2+^ entry at 10 μmol/L (≥17 cells/group). (*E*) Concentration-dependent inhibitory effects of CM_128 on the initial rate of Ca^2+^ entry.

To determine the effect of CM_128 on SOCE into isolated murine pancreatic acinar cells, thapsigargin was used to empty Ca^2+^ stores and initiate STIM-mediated ORAI pore formation, while maintaining cells in zero external Ca^2+^ until Ca^2+^ was re-introduced to enable SOCE.^1^, ^3^ Application of this protocol showed that CM_128 reduced SOCE markedly, at a lower dose than that of GSK-7975A (1 μmol/L) (Figure 3*A* and *B*); this same dose also was effective in significantly reducing necrotic cell death pathway activation by TLCS in these cells (Figure 3*C*). To confirm the effect of CM_128 on SOCE and to determine dose-dependency, cyclopiazonic acid was used to empty Ca^2+^ stores within murine pancreatic acinar cells^10^ (maintained in zero external Ca^2+^) to stimulate STIM-mediated ORAI opening. Upon reintroduction of external Ca^2+^ (1.8 mmol/L), the rate of Ca^2+^ entry showed concentration-dependent log proportionality, with the IC_50_ at approximately 0.7 μmol/L and no loss of effect at high concentrations (10 μmol/L) (Figure 3*D* and *E*).

### Effects of GSK-7975A on Experimental Acute Pancreatitis

**Supplementary Figure 2:**
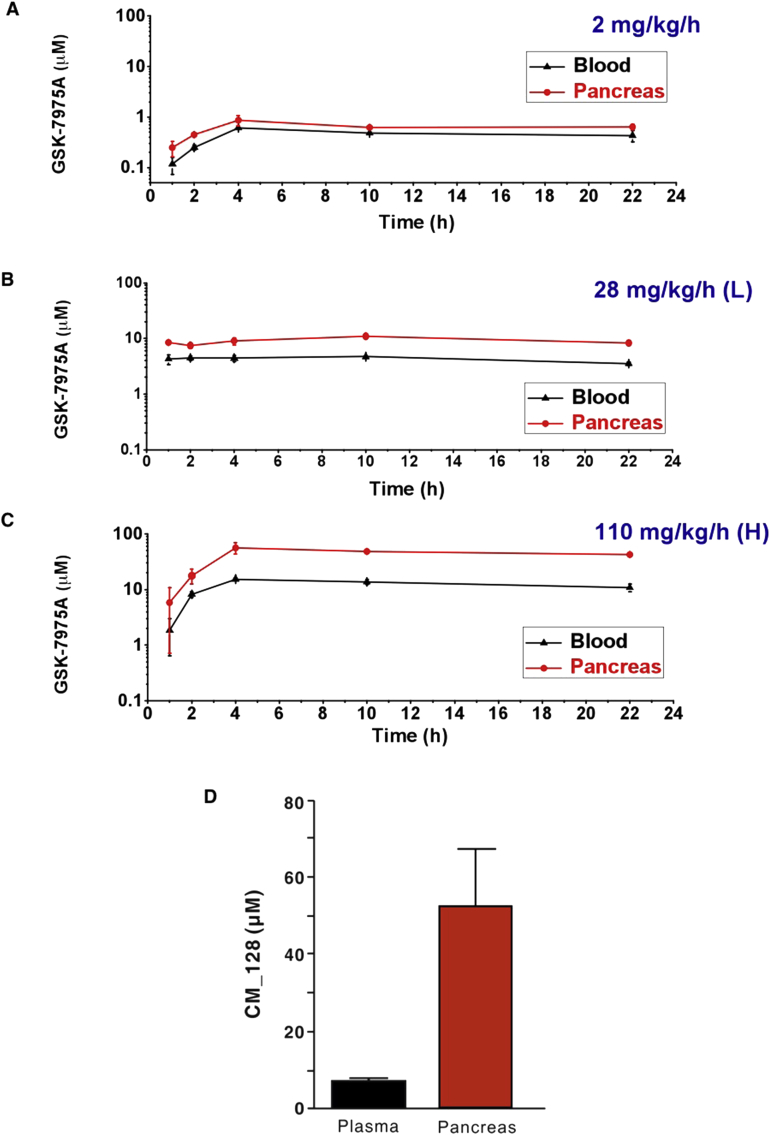
GSK-7975A given as prodrug GSK-6288B administered by a subcutaneous osmotic minipump can be delivered consistently to each mouse and maintained throughout the experimental period. There was no detectable GSK-6288B in the blood or pancreas, suggesting complete conversion into GSK-7975A. (*A*) Blood and pancreas levels of GSK-7975A after administration of 2 mg/kg/h GSK-6288B showing a steady state 4 hours after minipump implantation, when the mean concentrations in blood and pancreas were approximately 0.4 and approximately 0.6 μmol/L respectively. (*B*) Blood and pancreas levels of GSK-7975A at the (lower) dose of 28 mg/kg/h GSK 6288B (L) reached a steady state 1 hour after minipump implantation, when the mean concentrations in blood and pancreas were approximately 5 and approximately 10 μmol/L, respectively. (*C*) Blood and pancreas levels of GSK-7975A at the (higher) dose of 110 mg/kg/h GSK-6288B (H) reached a steady state 4 hours after minipump implantation, when the mean concentrations in blood and pancreas were approximately 15 and approximately 50 μmol/L, respectively. (*D*) Mean (±SEM) plasma and pancreas levels of CM_128 at 20 mg/kg sampling at the time point when drug efficacy was assessed, were approximately 10 and approximately 50 μmol/L, respectively.

To ensure consistent delivery of GSK-7975A in vivo we tested subcutaneous minipump administration of GSK-7975A against a background of CER-AP. Because of the modest aqueous solubility of GSK-7975A, we used a phosphate prodrug (GSK-6288B) that is cleaved rapidly in vivo to liberate GSK-7975A. Blood and pancreatic levels of GSK-7975A reached a steady state within 4 hours at all doses tested (Supplementary Figure 2). GSK-7975A at 28 (low) and 110 (high) mg/kg/h achieved steady-state blood concentrations of approximately 4.3 μmol/L and approximately 13.3 μmol/L, and pancreatic concentrations of approximately 8.9 μmol/L and approximately 49.3 μmol/L, respectively, with no detectable prodrug at all doses and time points.

**Figure 4:**
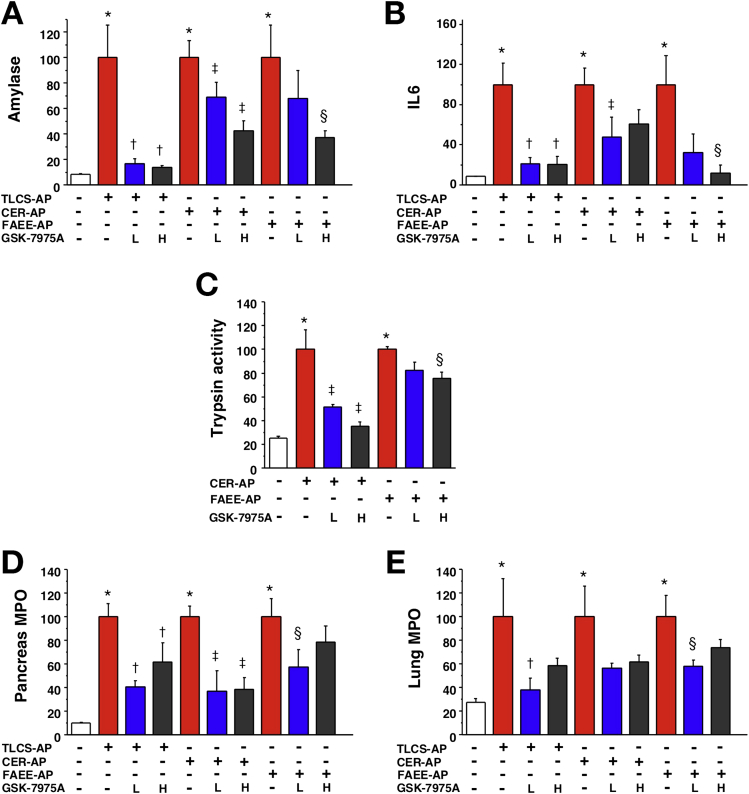
GSK-7975A markedly reduces all biochemical responses of TLCS-AP, CER-AP, and FAEE-AP. All models resulted in substantial increases of (*A*) serum amylase, (*B*) IL6, (*C*) pancreatic trypsin activity, (*D*) pancreatic activity, and (*E*) lung MPO activity. Subcutaneous osmotic minipump administration of GSK-7975A given as prodrug GSK-6288B at low (L) or high (H) doses significantly reduced all parameters, with a more marked reduction of serum amylase and IL6, pancreatic trypsin at the high dose (mean ± SEM ≥6 mice/group; *P < .05, control vs 3 models; ^†^*P* < .05 TLCS-AP vs TLCS-AP plus GSK-7975A; ^‡^*P* < .05, CER-AP vs CER-AP plus GSK-7975A; and ^§^*P* < .05, FAEE-AP vs FAEE-AP plus GSK-7975A).

**Figure 5:**
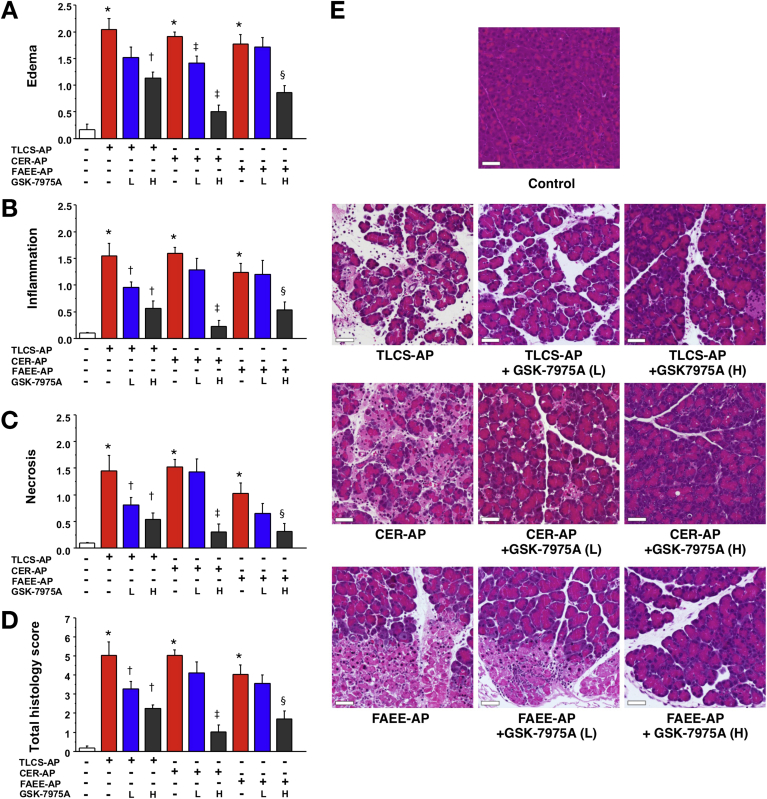
GSK-7975A markedly reduces pancreatic histopathology in TLCS-AP, CER-AP, and FAEE-AP. All models resulted in substantial increases in (*A*) edema, (*B*) inflammation, (*C*) necrosis, and (*D*) total histology score. Subcutaneous osmotic minipump administration of GSK-7975A given as prodrug GSK-6288B at low (L) or high (H) doses markedly reduced pancreatic damage, with more marked reduction at high dose (mean ± SEM ≥6 mice/group; *P < .05 control vs 3 models; ^†^*P* < .05, TLCS-AP vs TLCS-AP plus GSK-7975A; ^‡^*P* < .05, CER-AP vs CER-AP plus GSK-7975A; and ^§^*P* < .05, FAEE-AP vs FAEE-AP plus GSK-7975A). (*E*) Representative images showing normal pancreatic histology, typical histopathology from all 3 models, and typical histopathology from all 3 models after treatment with GSK-7975A at low (L) or high (H) doses (H&E; *scale bar*: 50 μm).

GSK-7975A was tested in 3 clinically representative mouse models of acute pancreatitis. TLCS-AP, which is representative of acute biliary pancreatitis from ampullary gallstone obstruction, was induced by pancreatic ductal infusion of TLCS^13^ and minipumps inserted 30 minutes later. At both doses GSK-7975A significantly reduced increases in serum amylase, IL6, and pancreatic MPO levels; lung MPO was reduced significantly by low dose only (Figure 4). There were consistent reductions in pancreatic edema, inflammatory cell infiltration, and acinar cell necrosis, with a marked reduction in overall histopathology score in the GSK-7975A–treated groups; inflammatory cell infiltration and histopathology score were reduced significantly more by the higher dose (Figure 5).

CER-AP is the most widely used model that is representative of acute pancreatitis induced by hyperstimulation,^14^ such as from anticholinesterase insecticides or *Tityus* species scorpion stings.^19^ In CER-AP, minipumps were inserted with the third of 7 cerulein injections. At both doses GSK-7975A significantly reduced the increases in serum amylase, pancreatic trypsin, and MPO levels, with the low dose resulting in significant reductions in IL6 and lung MPO levels (Figure 4). Pancreatic histopathology showed a trend toward a reduction in the low-dose group; at the high dose, there were significant marked reductions in all measures of pancreatic histopathology, approaching control levels (Figure 5).

FAEE-AP parallels acute alcoholic pancreatitis through in vivo formation of toxic ethanol metabolites.^15^ Minipumps were inserted in FAEE-AP at 1 hour after the second of 2 intraperitoneal injections of ethanol and palmitoleic acid. GSK-7975A reduced the increases in all parameters, with pancreatic and lung MPO levels significantly reduced at the low dose; serum amylase level, IL6 level, pancreatic trypsin level, and histopathology were reduced significantly at the high dose (Figures 4 and 5). There were significantly greater reductions in edema, inflammation, and the overall histopathology score at the high dose, with levels of necrosis approaching control levels (Figure 5). In all models, low-dose GSK-7975A was generally as effective as the high dose in reducing IL6, which contributes to lung injury and lethality,^20^ and MPO. These data are consistent with the lower IC_50_ of GSK-7975A on ORAI channel SOCE in leukocytes (∼1 μmol/L for T lymphocytes)^7^, ^8^ than in pancreatic acinar cells (3.4 μmol/L).^3^

### Effects of CM_128 on Experimental Acute Pancreatitis

**Figure 6:**
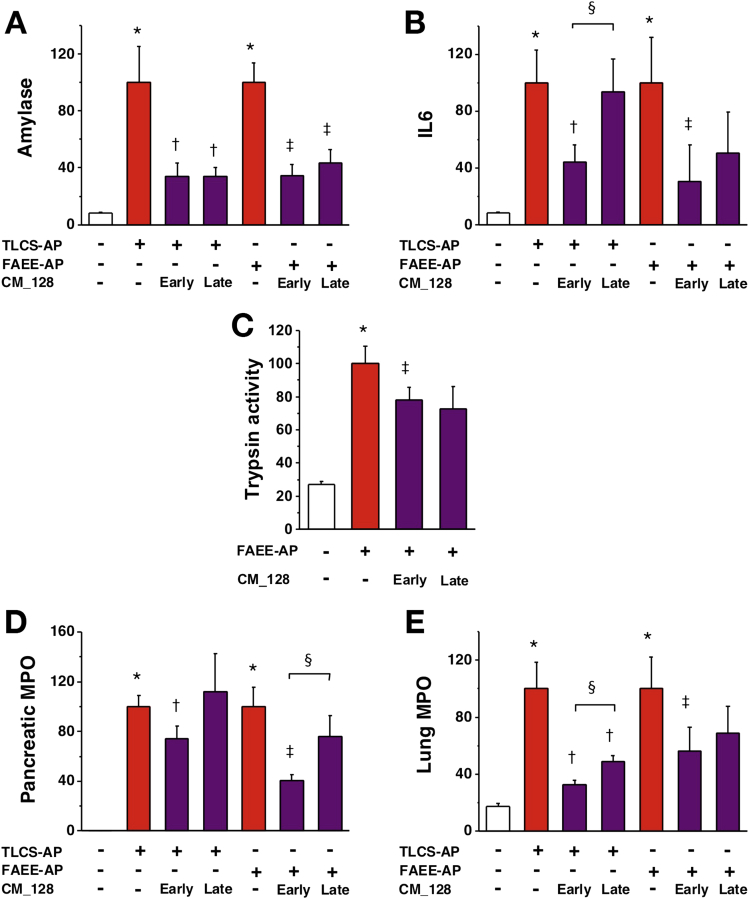
CM_128 markedly reduces all biochemical responses of TLCS-AP and FAEE-AP. Two models resulted in substantial increases of (*A*) serum amylase, (*B*) IL6, (*C*) pancreatic trypsin activity, (*D*) pancreatic activity, and (*E*) lung MPO activity. Intraperitoneal administration of CM_128 at 20 mg/kg given at 1 hour after disease induction (early) and 6 hours after (late) significantly reduced all parameters, with more marked reduction of IL6, pancreatic activity, and lung MPO activity when CM_128 was administered early (mean ± SEM ≥6 mice/group; *P < .05, control vs 2 models; ^†^*P* < .05 TLCS-AP vs TLCS-AP plus CM_128; ^‡^*P* < .05, FAEE-AP vs FAEE-AP plus CM_128; and ^§^*P* < .05, CM_128 early vs late).

**Figure 7:**
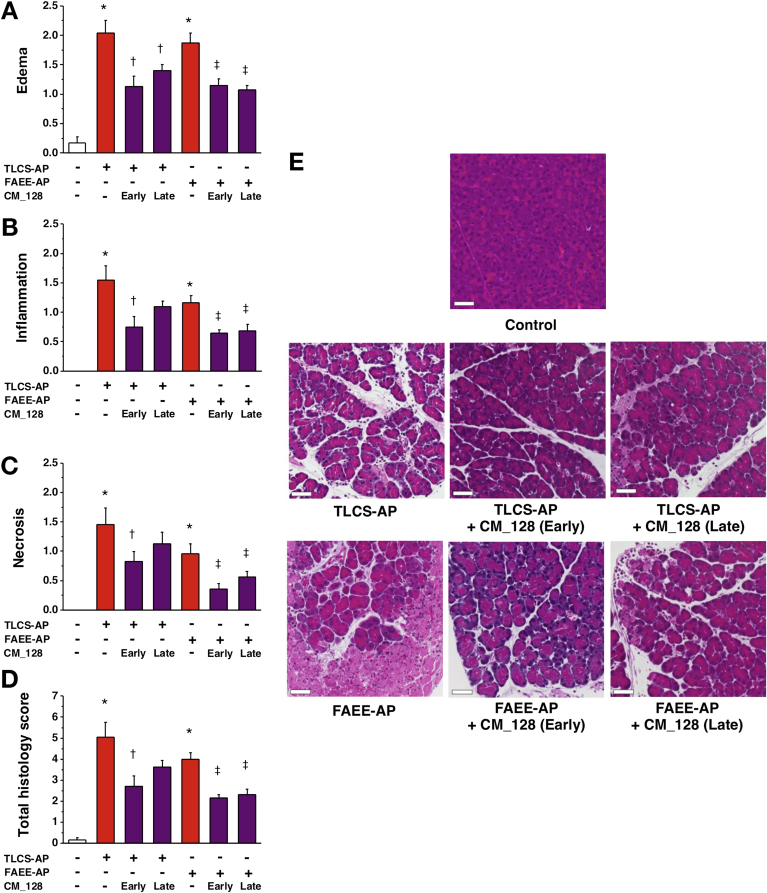
CM_128 markedly reduces pancreatic histopathology in TLCS-AP and FAEE-AP. Both models resulted in substantial increases in (*A*) edema, (*B*) inflammation, (*C*) necrosis, and (*D*) total histology score. Intraperitoneal administration of CM_128 at 20 mg/kg given at 1 hour after disease induction (early) and 6 hours after (late) significantly reduced all parameters, with a more marked reduction when CM_128 was administered early (mean ± SEM ≥6 mice/group; *P < .05, control vs 2 models; ^†^*P* < .05 TLCS-AP vs TLCS-AP plus CM_128; ^‡^*P* < .05, FAEE-AP vs FAEE-AP plus CM_128; and ^§^*P* < .05, CM_128 early vs late). (*E*) Representative images showing normal pancreatic histology, typical histopathology from 2 models, and typical histopathology from 2 models after treatment with CM_128 early and late after disease induction (H&E; *scale bar*: 50 μm).

**Supplementary Figure 3:**
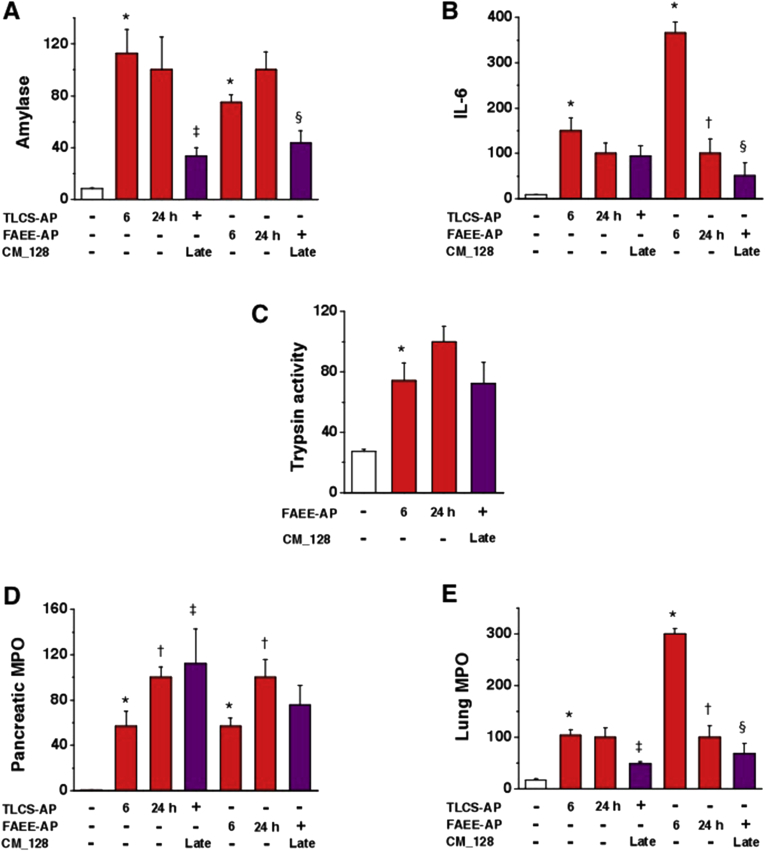
CM_128 administered from 6 hours after disease induction (late) markedly reduced biochemical responses in TLCS-AP and FAEE-AP. Two models at 6 and 24 hours resulted in substantial increase of (*A*) serum amylase, (*B*) IL6, (*C*) pancreatic trypsin activity, (*D*) pancreatic activity, and (*E*) lung MPO activity, with more marked increase of IL6 and lung MPO activity at 6 hours, but of pancreatic trypsin and MPO activity at 24 hours. Intraperitoneal administration of CM_128 at 20 mg/kg late significantly reduced all parameters from levels at 6 hours (mean ± SEM ≥6 mice/group; *P < .05, control vs 2 models at 6 h; ^†^*P* < .05 2 models at 6 vs 24 h; ^‡^*P* < .05, TLCS-AP at 6 h vs TLCS-AP plus CM_128; ^§^*P* < .05, FAEE-AP at 6 h vs FAEE-AP plus CM_128).

**Supplementary Figure 4:**
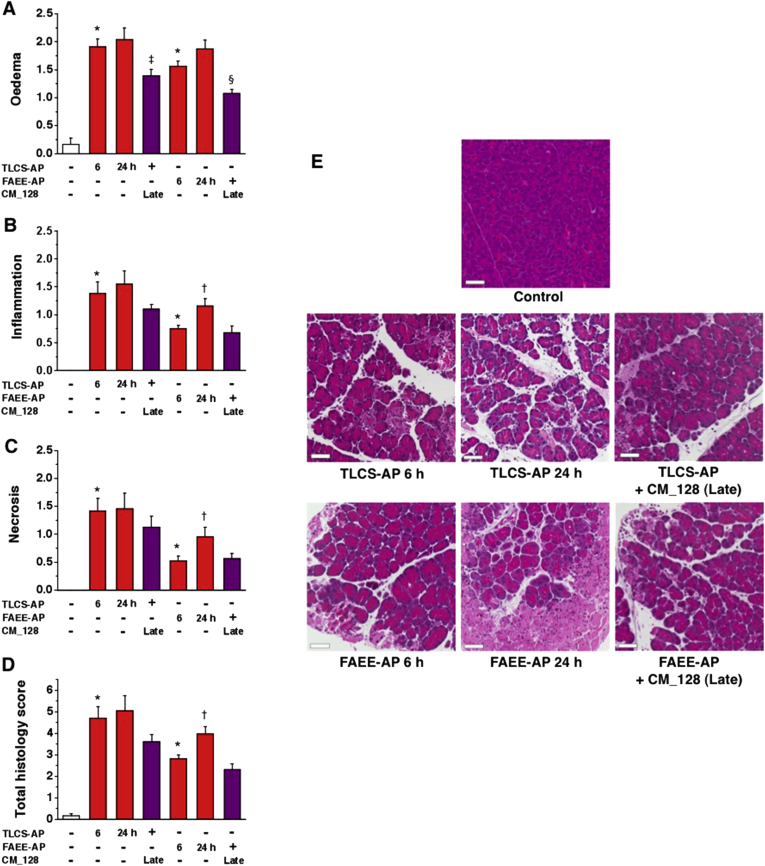
CM_128 administered from 6 hours after disease induction (late) markedly reduced pancreatic histopathology in TLCS-AP and FAEE-AP. Two models at 6 and 24 hours resulted in substantially progressive increases in (*A*) edema, (*B*) inflammation, (*C*) necrosis, and (*D*) total histology score, with more marked increase of all scores at 24 hours. Intraperitoneal administration of CM_128 at 20 mg/kg from 6 hours after disease induction significantly reduced edema, but not inflammation, necrosis, or total histology scores at 6 hours (mean ± SEM ≥6 mice/group; *P < .05, control vs 2 models at 6 hours; ^†^*P* < .05 2 models at 6 vs 24 hours; ^‡^*P* < .05, TLCS-AP at 6 h vs TLCS-AP plus CM_128; ^§^*P* < .05, FAEE-AP at 6 h vs FAEE-AP plus CM_128). (*E*) Representative images showing normal pancreatic histology, typical histopathology from 2 models at 6 and 24 hours, and typical histopathology from 2 models after treatment with CM_128 administered late after disease induction (H&E; *scale bar*: 50 μm).

**Supplementary Figure 5:**
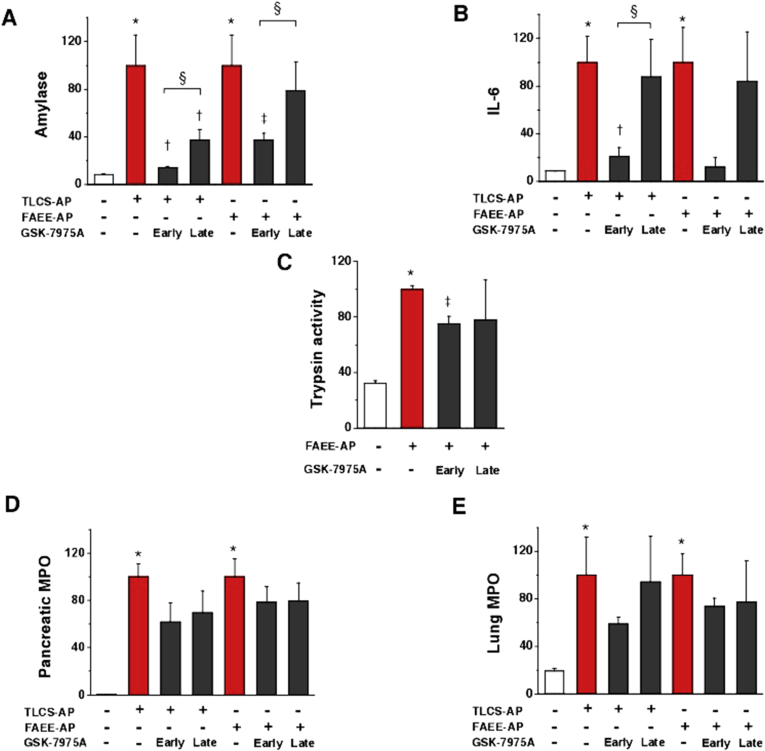
GSK-7975A administered from 6 hours after disease induction (late) less effectively reduced biochemical responses of TLCS-AP and FAEE-AP. Two models resulted in substantial increases of (*A*) serum amylase, (*B*) IL6, (*C*) pancreatic trypsin activity, (*D*) pancreatic activity, and (*E*) lung MPO activity. Subcutaneous osmotic minipump administration of GSK-7975A given as prodrug GSK-6288B at high dose administered from a late time point was less protective than when given early (mean ± SEM ≥6 mice/group; *P < .05, control vs 2 models; ^†^*P* < .05 TLCS-AP vs TLCS-AP plus GSK-7975A; ^‡^*P* < .05, FAEE-AP vs FAEE-AP plus GSK-7975A; and ^§^*P* < .05, GSK-7975A early vs GSK-7975A late).

**Supplementary Figure 6:**
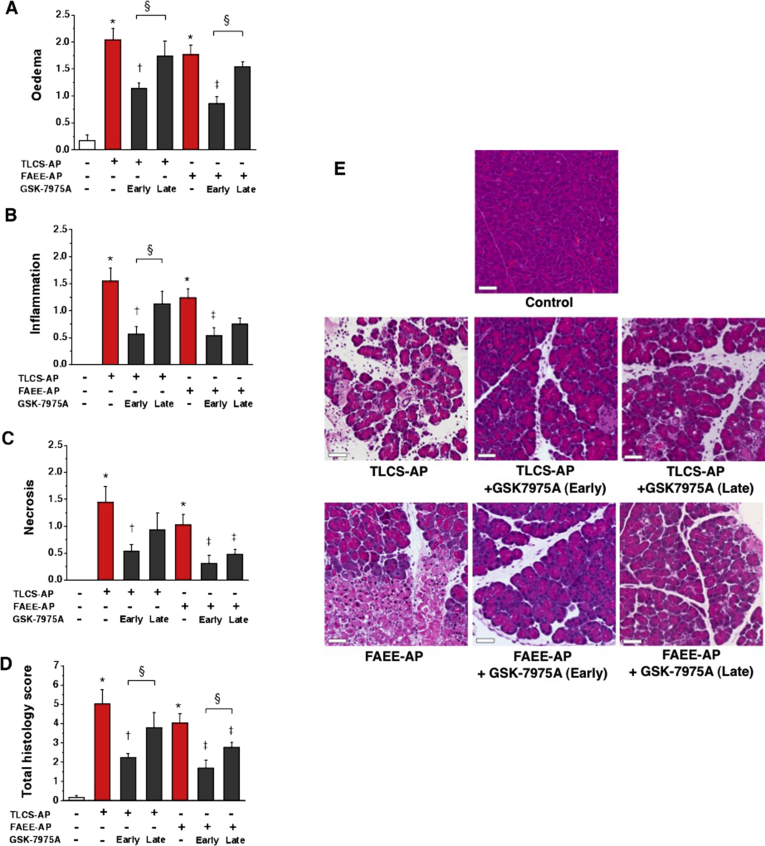
GSK-7975A administered from 6 hours after disease induction (late) less effectively reduced pancreatic histopathology in TLCS-AP and FAEE-AP. Two models resulted in substantial increases in (*A*) edema, (*B*) inflammation, (*C*) necrosis, and (*D*) total histology score. Subcutaneous osmotic minipump administration of GSK-7975A given as prodrug GSK-6288B at high dose administered from a late time point was less effective that when begun early (mean ± SEM ≥6 mice/group; *P < .05, control vs 2 models; ^†^*P* < .05 TLCS-AP vs TLCS-AP plus GSK-7975A; ^‡^*P* < .05, FAEE-AP vs FAEE-AP plus GSK-7975A, and ^§^*P* < .05, GSK-7975A early vs GSK-7975A late). (*E*) Representative images showing normal pancreatic histology, typical histopathology from 2 models, and typical histopathology from 2 models after treatment with GSK-7975A administered early and late after disease induction (H&E; *scale bar*: 50 μm).

Preliminary in vivo experiments indicated that CM_128 has a significantly longer half-life than GSK-7975A, and is suitable for intraperitoneal dosing every 12 hours to achieve sustained blood levels with more than 99% bound (free fraction in murine plasma, 0.33%; when added to human plasma, 0.16%). Because our work with high-dose GSK-7975A showed greater efficacy in vivo than with low-dose GSK-7975A, and in vitro data obtained with CM_128 did not suggest loss of efficacy at high concentrations (10 μmol/L), we administered 20 mg/kg CM_128 every 12 hours to test the efficacy of this agent in TLCS-AP and FAEE-AP. We also determined the relative efficacy of CM_128 administered either 1 or 6 hours after induction of either model of acute pancreatitis. CM_128 begun 1 hour after disease induction significantly reduced all parameters of both TLCS-AP and FAEE-AP, including all local and systemic biochemical, immunologic, and histopathologic measures (Figures 6 and 7). CM_128 begun 6 hours after disease induction was less effective across a broad range of parameters (Figures 6 and 7), significantly so for IL6 (TLCS-AP), pancreatic MPO (FAEE-AP), and lung MPO (TLCS-AP), although significant reductions still were seen in amylase (TLCS-AP and FAEE-AP), lung MPO (TLCS-AP), edema (TLCS-AP and FAEE-AP), inflammation (FAEE-AP), necrosis (FAEE-AP), and total histopathology score (FAEE-AP). To determine the extent to which disease was established at 6 hours after disease induction, and the effect of CM_128 begun at that time, all parameters were assessed at 6 hours and compared with values at 24 hours. These data showed that by 24 hours there was no significant improvement of parameters as measured at 6 hours as a result of CM_128 administration begun at 6 hours (Supplementary Figures 3 and 4), confirming delay in therapy to be disadvantageous; although CM_128 appeared to prevent these parameters from increasing. To further explore the effect of delay in dosing, the effect of high-dose GSK-7975A on disease responses also was tested at 1 and 6 hours after induction. Similar to CM_128, GSK-7975A begun 6 hours after disease induction was less effective across a broad range of parameters (Supplementary Figures 5 and 6), significantly so for amylase (TLCS-AP and FAEE-AP), IL6 (TLCS-AP), edema (TLCS-AP and FAEE-AP), inflammatory infiltrate (TLCS-AP), and total histopathology score (TLCS-AP and FAEE-AP).

## Discussion

We found GSK-7975A and the new molecular entity CM_128 to inhibit toxin-induced SOCE into murine and human pancreatic acinar cells in a concentration-dependent manner, exceeding more than 90% block of relative control values in some protocols. We also found both GSK-7975A and CM_128 to reduce significantly the necrotic cell death pathway activation in murine and human pancreatic acinar cells exposed to TLCS, which induces acute pancreatitis in vivo.^13^, ^14^ Although effects of GSK-7975A have been described on thapsigargin- and palmitoleic acid ethyl ester–induced murine pancreatic acinar SOCE,^3^ our study found GSK-7975A to have a similarly critical effect on TLCS- and CCK-induced murine pancreatic acinar SOCE, as well as thapsigargin-induced human pancreatic acinar SOCE and TLCS-induced human pancreatic acinar necrotic cell death pathway activation. CM_128 showed a higher potency (IC_50_ ∼0.1 μmol/L from ORAI1/STIM1-transfected HEK 293 cell patch-clamp data), and unlike GSK-7975A, no loss of efficacy at high doses. Comprehensive in vivo evaluation using 3 diverse, clinically representative models of acute pancreatitis^14^ with prior pharmacokinetic assessment showed the validity of SOCE inhibition as a therapeutic approach. Thus, administration of either compound within 1 hour after disease induction was markedly effective across a representative range of local and systemic biochemical, immunologic, and histologic disease responses.

Our novel human data support the potential applicability of SOCE inhibition as a treatment for clinical acute pancreatitis. Both GSK-7975A and CM_128 blocked SOCE promptly, shown here to result in complete block of human ORAI1 by CM_128. Although an action on other ORAI channels cannot be excluded and could be desirable, ORAI1 is the primary channel for SOCE into pancreatic acinar cells,^3^, ^5^ blocked by both compounds. ORAI channels also contribute to inflammatory cell responses, including neutrophil migration and activation^21^; inhibition of innate immune responses significantly reduces the severity of experimental acute pancreatitis,^22^ thus there may be a contribution here from ORAI inhibition of immune cells. Nevertheless, although knockout of ORAI1/STIM1 SOCE inhibits neutrophil functions, it does not prevent all functions,^21^ so the primary contribution of ORAI blockade in our experiments is likely to have been in the pancreas. Furthermore, because SOCE inhibition for clinical acute pancreatitis would necessarily be short term, inhibition of the adaptive immune system^21^ also would be short term. ORAI blockade has less effect on other cell types in which ORAI channels have a less prominent role, such as electrically excitable cells in which other ion channels (eg, nonselective cation channels) have a larger role in Ca^2+^ entry.^23^ Nonselective cation channels, however, permit limited SOCE into pancreatic acinar cells^3^, ^10^ that could sustain essential Ca^2+^ entry.^23^ Without such Ca^2+^ entry, continued activation of the plasma membrane Ca^2+^-adenosine triphosphatase pump upon secretagogue- or toxin-mediated release of Ca^2+^ from intracellular stores could deplete these stores to deleterious levels, inducing or exacerbating endoplasmic reticulum stress.^24^

Measurement of blood and tissue levels of GSK-7975A after induction of experimental acute pancreatitis established an appropriate dosing regimen (110 mg/kg/h via minipump) for maximum effect, at a steady state of 10–15 μmol/L in blood and approximately 50 μmol/L in the pancreas, with less than 10% free GSK-7975A. Our cell data indicated that at 50 μmol/L, GSK-7975A had no loss of effect, and the concentration of free compound in vivo was significantly lower. At this dose, however, GSK-7975A was highly effective in reducing all measures of disease response in 3 clinically representative models of acute pancreatitis (TLCS-AP, CER-AP, and FAEE-AP), and more so than at a lower dose (28 mg/kg/h). CM_128, with higher potency than GSK-7975A but higher levels of plasma and tissue binding, was tested at 20 mg/kg given every 12 hours via intraperitoneal injection in TLCS-AP and FAEE-AP, representative of gallstone and alcoholic acute pancreatitis,^14^, ^15^ the most common forms of the disease.^4^ This resulted in CM_128 levels greater than 7 μmol/L in blood and approximately 50 μmol/L in the pancreas 11 hours after the last dose, levels that were highly effective in reducing all disease parameters. These data provide robust confirmation of the hypothesis that cytosolic Ca^2+^ overload is a critical trigger of acute pancreatitis.^25^

Both compounds were administered after disease induction to model treatment of clinical acute pancreatitis, but a delay in administration of either compound to 6 hours after disease induction resulted in diminished efficacy, dependent on the end point measured and the model used. Although biological time courses including that of acute pancreatitis are longer in human beings than in mice,^4^, ^14^, ^26^ with pancreatic necrosis typically detected within days rather than hours,^4^, ^27^ human pancreatic acinar necrotic cell death pathway activation may begin in clinical acute pancreatitis at an early stage after disease onset, shown here in mouse models within 6 hours of onset. Door-to-needle times of less than 60 minutes are established guidelines for patients with acute myocardial infarction (30 min)^28^ and acute ischemic stroke (60 min),^29^ making every second count, with national and international quality-improvement initiatives underway toward fully achieving these.^30^ Although pancreatic necrosis has a less rapid time course and is not the result of major arterial occlusion,^4^ the translational implication of our work is that door-to-needle time is an important issue in administration of any treatment for acute pancreatitis that targets the pathogenesis of pancreatic injury, which drives the disease. Previously, clinical trials of treatments for acute pancreatitis “enriched” recruitment with patients predicted to have severe disease (often with recruitment up to 72 h after admission),^4^, ^31^ which delays the initiation of therapy. Furthermore, the expansion of disease categories from the original Atlanta Classification (mild and severe)^32^ into the revised Atlanta (mild, moderate, and severe)^33^ and Determinants-Based (mild, moderate, severe, and critical)^34^ classification, further complicates patient selection from among these potentially overlapping subgroups. To minimize door-to-needle time, a quicker and more accurate approach to the selection of patients is required for trials of any therapy, such as that offered here with ORAI inhibition by CM_128, a novel molecular entity currently undergoing preclinical toxicologic evaluation before phase I trials.

## Supplementary Materials and Methods

### Measurements of GSK-7975A and CM_128 In Vivo

Sampling of GSK-7975A was at 1, 2, 4, 10, and 22 hours after osmotic minipump insertion from 3 mice/time point. Immediately after humane killing, blood was collected into a heparinized tube, diluted 1:1 with sterile water, and the pancreas was removed and homogenized in 2 mL Covaris tubes containing ceramic beads. Standards and study samples (50 μL from blood and 100 μL from pancreas) were extracted by protein precipitation and centrifuged. Supernatants were dried under heated nitrogen (40°C). Levels of GSK-7975A and GSK-6288B were determined by LC-MS/MS (API4000 with Jasco X-LC and Ascentis express C18 column), as were levels of CM_128 in plasma and pancreas (Varian 500-MS with Varian 212 LC and Phenomenex C8 column), sampling from 3 mice at the same time point when drug efficacy was assessed.

### Protein Binding of GSK-7975A and CM_128

The protein binding of GSK-7975A in the blood and pancreas was determined at 1 μg/mL using a dialysis Teflon block (HTDialysis, LLC, Gales Ferry, CT) with dialysis membrane strips and a rapid equilibrium dialysis device (Thermo Scientific), respectively. The protein binding of CM_128 in plasma (from Bioreclamation, Inc, Westbury, NY) was determined at 30 μmol/L or 50 μmol/L by equilibrium dialysis using a high-throughput dialysis Teflon block (HTDialysis, LLC) and dialysis membrane strips. Blood was collected into a heparin precoated tube and diluted 1:1 with phosphate-buffered saline (pH 7.4). Pancreas was homogenized in 500 μL phosphate-buffered saline, diluted 1:10. Blood, pancreas, homogenate, and plasma were dialyzed against phosphate-buffered saline in a buffer chamber, shaking gently at 37°C for 5 hours (blood), 4 hours (pancreas homogenate), and overnight (plasma). After equilibrium, samples from both the sample chamber and the buffer chamber of the rapid equilibrium dialysis plate were collected in a matrix-match manner and analyzed by liquid chromatography–mass spectrometry (AP14000; Applied Biosystems; or Varian 500-MS with Varian 212 LC and Phenomenex C8 column). Experiments were performed in triplicate. The percentage of GSK-7975A bound was calculated by the following equation: % bound= ([GSK-7975A]_a_ - [GSK-7975A]_b_)/[GSK-7975A]_a_ ×100%, where [GSK-7975A]_a_ was the concentration of GSK-7975A in the sample chamber and [GSK-7975A]_b_ was the concentration of GSK-7975A in the buffer chamber. The same equation was applied to calculate CM_128 binding.
